# ABCG2 Is Overexpressed on Red Blood Cells in Ph-Negative Myeloproliferative Neoplasms and Potentiates Ruxolitinib-Induced Apoptosis

**DOI:** 10.3390/ijms22073530

**Published:** 2021-03-29

**Authors:** Ralfs Buks, Mégane Brusson, Sylvie Cochet, Tatiana Galochkina, Bruno Cassinat, Ivan Nemazanyy, Thierry Peyrard, Jean-Jacques Kiladjian, Alexandre G. de Brevern, Slim Azouzi, Wassim El Nemer

**Affiliations:** 1 BIGR, UMR_S1134, Inserm, Université de Paris, F-75015 Paris, France; ralfs.buks@inserm.fr (R.B.); megane.brusson@institutimagine.org (M.B.); sylvie.cochet@inserm.fr (S.C.); tatiana.galochkina@inserm.fr (T.G.); tpeyrard@ints.fr (T.P.); alexandre.debrevern@univ-paris-diderot.fr (A.G.d.B.); slim.azouzi@inserm.fr (S.A.); 2 Institut National de la Transfusion Sanguine, F-75015 Paris, France; 3 Laboratoire d’Excellence GR-Ex, F-75015 Paris, France; bruno.cassinat@aphp.fr (B.C.); jean-jacques.kiladjian@aphp.fr (J.-J.K.); 4 IRSL, U1131, INSERM, Université de Paris, F-75010 Paris, France; 5 Hôpital Saint-Louis, Laboratoire de Biologie Cellulaire, AP-HP, F-75010 Paris, France; 6 Platform for Metabolic Analyses, Structure Fédérative de Recherche Necker, INSERM US24/CNRS UMS 3633, F-75015 Paris, France; ivan.nemazanyy@inserm.fr; 7 Centre National de Référence Pour les Groupes Sanguins, F-75011 Paris, France; 8 Centre d’Investigations Cliniques, Hôpital Saint-Louis, Université de Paris, F-75010 Paris, France; 9 UMR 7268, 27 Boulevard Jean Moulin, F-13005 Marseille, France

**Keywords:** polycythemia vera, JAK2V617F, red blood cells, ABCG2, ruxolitinib, hydroxyurea

## Abstract

Myeloproliferative neoplasms (MPNs) are a group of disorders characterized by clonal expansion of abnormal hematopoietic stem cells leading to hyperproliferation of one or more myeloid lineages. The main complications in MPNs are high risk of thrombosis and progression to myelofibrosis and leukemia. MPN patients with high risk scores are treated by hydroxyurea (HU), interferon-α, or ruxolitinib, a tyrosine kinase inhibitor. Polycythemia vera (PV) is an MPN characterized by overproduction of red blood cells (RBCs). ABCG2 is a member of the ATP-binding cassette superfamily transporters known to play a crucial role in multidrug resistance development. Proteome analysis showed higher ABCG2 levels in PV RBCs compared to RBCs from healthy controls and an additional increase of these levels in PV patients treated with HU, suggesting that ABCG2 might play a role in multidrug resistance in MPNs. In this work, we explored the role of ABCG2 in the transport of ruxolitinib and HU using human cell lines, RBCs, and in vitro differentiated erythroid progenitors. Using stopped-flow analysis, we showed that HU is not a substrate for ABCG2. Using transfected K562 cells expressing three different levels of recombinant ABCG2, MPN RBCs, and cultured erythroblasts, we showed that ABCG2 potentiates ruxolitinib-induced cytotoxicity that was blocked by the ABCG2-specific inhibitor KO143 suggesting ruxolitinib intracellular import by ABCG2. In silico modeling analysis identified possible ruxolitinib-binding site locations within the cavities of ABCG2. Our study opens new perspectives in ruxolitinib efficacy research targeting cell types depending on ABCG2 expression and polymorphisms among patients.

## 1. Introduction

Myeloproliferative neoplasms (MPNs) are a group of hematological disorders characterized by clonal expansion of abnormal hematopoietic stem cells leading to excessive proliferation of one or more myeloid cell lines in the bone marrow. Polycythemia vera (PV) and essential thrombocythemia (ET) are types of Philadelphia chromosome (Ph)-negative MPNs characterized by overproduction of red blood cells (RBCs) and platelets, respectively. Over 95% of PV patients and 60% of ET patients display the V617F mutation in the Janus kinase 2 (JAK2) tyrosine kinase [1]. In normal conditions, erythroid progenitors proliferate and differentiate through the erythropoietin (Epo)-stimulated JAK/STAT signaling pathway. However, in the presence of JAK2V617F, the JAK/STAT signaling pathway continuously actively leads to erythroid proliferation in the absence of Epo [2,3,4,5]. High risk of thrombosis together with progression to myelofibrosis and leukemia represent the main complications of this disease. Patients with high prognostic risk scores are treated by cytoreductive therapy, such as hydroxyurea (HU) or interferon-α (IFN-α) or by ruxolitinib, a JAK1/JAK2 inhibitor.

The group of ATP-binding cassette (ABC) superfamily transporters are known to play a crucial role in multidrug resistance development due to their drug efflux capabilities in cancer cells. The superfamily member ABCG2, also known as the breast cancer resistance protein (BCRP), protects tissues against xenobiotics and affects the pharmacokinetics of drugs [6]. Increased protein levels of ABCG2 and ABCB1, another ABC transporter, are linked with increased resistance to numerous drugs including tyrosine kinase inhibitors (TKIs), such as imatinib and nilotinib [7]. Despite ruxolitinib being used in MPN treatment, the current knowledge of its transport into the intracellular compartment remains poor. It has been suggested that ruxolitinib is either a substrate or allosteric inhibitor of ABCB1 and that it does not alter ABCG2 function [8].

Transcriptomic analyses have suggested an increase in ABCG2 levels in maturing cells during erythropoiesis [9,10]. Our PV RBC proteome analysis using mass spectrometry showed higher ABCG2 levels in PV RBCs compared to RBCs from healthy controls [11] and an additional increase of these levels in PV patients treated with HU [12], suggesting that ABCG2 might play a role in multidrug resistance in MPNs.

In this study, we investigated the potential role of ABCG2 in the transport of ruxolitinib and HU. We used a fast kinetics approach (stopped flow) to study HU transport by ABCG2 in RBCs and developed cytotoxicity and apoptosis assays to test ruxolitinib transport by ABCG2 using a cell line model, as well as RBCs and in vitro differentiated erythroblasts. We used LC–MS to study direct ruxolitinib transport by ABCG2 in a cell line model and in silico analysis to identify ruxolitinib-binding site locations. Our data suggest ruxolitinib import by ABCG2 in the erythroid lineage suggesting a potential role for this transporter in the response to ruxolitinib treatment in MPN patients.

## 2. Results

### 2.1. HU Treatment Increases ABCG2 Levels on the RBC Surface and on the Surface of ABCG2-Expressing Cell Lines

**Figure 1:**
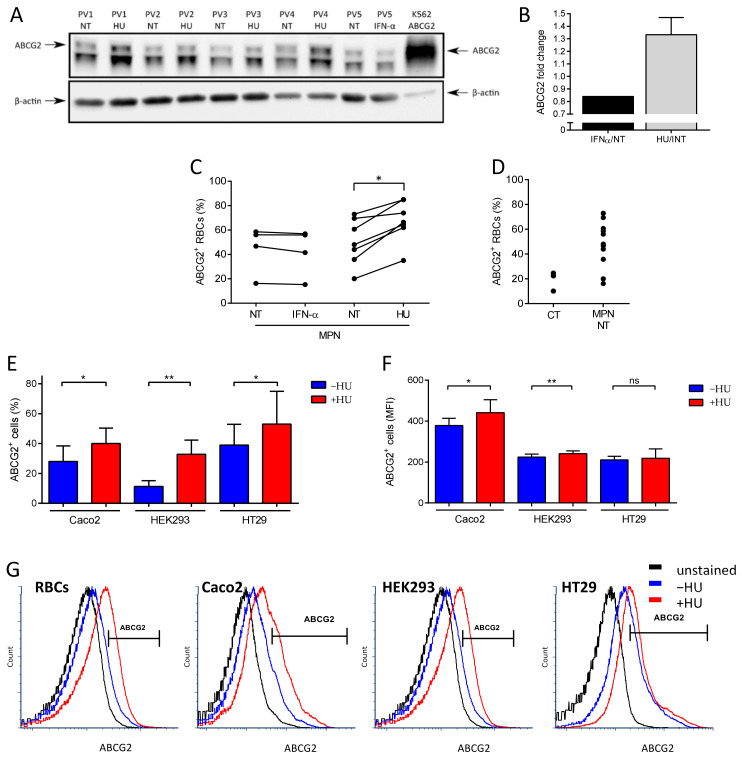
Hydroxyurea treatment increases ABCG2 levels on the RBC surface and on the surface of ABCG2-expressing cell lines. (**A**) SDS-PAGE immunoblots of ABCG2 expression on RBC membranes from four PV patients before and during HU treatment and one PV patient before and during IFN-α treatment. A K562 cell line transfected with ABCG2 was used as the positive control. The lower band in the ABCG2 panel is a non-specific cross-reacting protein. Beta-actin served as the loading control. (**B**) ABCG2 protein fold change on five PV RBC membranes from patients during HU (black) or IFN-α treatment (grey) displayed as the means with SDs. Flow cytometry analysis of ABCG2 expression presented as percentage of ABCG2-positive (ABCG2^+^) RBCs of (**C**) four MPN patients before and during IFN-α treatment, seven MPN patients before and during HU treatment, (**D**) three healthy individuals (CT), and 11 non-treated (NT) MPN patients. Wilcoxon test, * *p* = 0.0156. (**E**) Flow cytometry analysis of ABCG2 expression on Caco2, HEK293, and HT29 cell lines presented as percentage of ABCG2-positive (ABCG2^+^) cells and (**F**) MFI of ABCG2-positive (ABCG2^+^) cells. Data are presented as the means with SDs, *N* = 6 (Caco2), *N* = 4 (HEK293, HT29); paired *t*-test, ns – not significant, * *p* < 0.05, ** *p* < 0.01. (**G**) Representative flow cytometry plots of ABCG2 expression on MPN RBCs, Caco2, HEK293, and HT29 cells. Unstained (black), before (blue) and during HU treatment (red).

To validate our previously published mass spectrometry results, we performed a longitudinal analysis to compare ABCG2 expression in RBCs of MPN patients before and during HU or IFN treatments. SDS-PAGE immunoblot analysis showed a 33% increase (SD = 11%) of ABCG2 levels during HU treatment while they remained constant under IFN-α treatment (Figure 1A,B). This increase was further confirmed and characterized by flow cytometry analyses, which showed higher percentages of ABCG2-positive RBCs in patients treated with HU and no difference in those treated with IFN-α (Figure 1C). Moreover, flow cytometry analyses showed a strong trend of higher percentages of ABCG2-positive RBCs in MPN patients prior to any treatment compared to healthy individuals (Figure 1D, *p* = 0.06) further validating our mass spectrometry data from untreated patients [11]. To further assess the role of HU in ABCG2 overexpression, we used three ABCG2 expressing human cell lines and treated them with HU in vitro. These cell lines were: human epithelial colorectal adenocarcinoma cells (Caco2), human embryonic kidney cells (HEK293), and human epithelial cells from colon cancer (HT29). Flow cytometry analyses showed higher percentages of ABCG2-positive cells in the three cell lines after incubation with HU and increased mean fluorescence intensity (MFI) of ABCG2 in Caco2 and HEK293 cells in the presence of HU, indicating that HU-induced ABCG2 overexpression was not restricted to the erythroid lineage (Figure 1E–G).

### 2.2. HU Is not a Substrate of ABCG2

**Figure 2:**
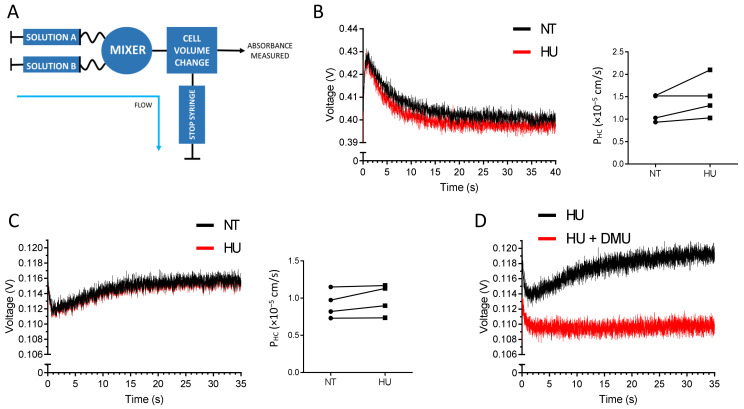
HU is not a substrate of ABCG2. (**A**) Schematic representation of the stopped-flow experimental setting. The RBC volume is measured in volts, and the cell volume dynamics is calculated as P_HC_ (10^−5^ cm/s). (**B**) Representative HU import gradient (left panel) and HU entry kinetics (right panel) from four PV patients’ RBCs before HU treatment (black) and after HU treatment (red). Solution A: RBC suspension in PBS; Solution B: PBS + 100 mM HU. (**C**) Representative HU export gradient (left panel) and HU export kinetics (right panel) from four PV patients’ RBCs before HU treatment (black) and after HU treatment (red). Solution A: RBC suspension in PBS + 100 mM HU; Solution B: PBS. (**D**) HU transport gradient upon the blockage of UT-B by DMU. Solution A: RBCs in PBS + 100 mM HU with (red) or without (black) 15 mM DMU; solution B: PBS with (red) or without (black) 15 mM DMU.

The bidirectional urea transporter B (UT-B) functions as a channel for urea and water, resulting in rapid cell volume increase when urea is imported [13,14,15,16]. HU is known to be transported by UT-B [17]. The UT-B levels on RBC membranes are similar between healthy individuals and PV patients, and HU treatment does not modify UT-B expression [11,12]. The increased expression of ABCG2 by HU suggested that the drug could be a potential substrate of the transporter which would export it from the intracellular compartment to the extracellular compartment in order to detoxify the cell. To test this hypothesis, we used a fast kinetics approach measuring cell volume changes by stopped flow, as such changes occur within a very short time frame (Figure 2A). Gradients of HU import and export kinetics were measured in RBC samples from four PV patients before and after HU treatment. HU import kinetics were measured by mixing RBC suspensions with a buffer containing HU (Figure 2B). In contrast, HU export kinetics were measured by mixing an RBC suspension containing HU with a buffer alone (Figure 2C). As expected, HU was imported and exported in all RBC samples (Figure 2B,C). Despite the high increase in ABCG2 levels on the RBC surface after HU treatment for all of the four patients, there was a slight but not significant difference of the import kinetics, and no difference in the export kinetics before and after HU (Figure 2B,C). To ensure that ABCG2 was not involved in HU transport, we measured export kinetics in RBCs preincubated with dimethylurea (DMU), a specific UT-B inhibitor [17]. The HU transport was fully blocked by DMU indicating that UT-B was the only HU transporter on the surface of RBCs and that ABCG2 was not transporting HU (Figure 2D). Altogether, our data indicate that HU is not a substrate of ABCG2.

### 2.3. ABCG2 Inhibition Reduces Ruxolitinib-Induced PS Exposure on MPN RBCs

**Figure 3:**
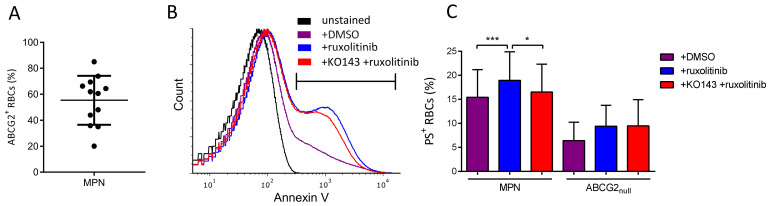
ABCG2 inhibition reduces ruxolitinib-induced PS exposure on MPN RBCs. (**A**) Flow cytometry analysis of ABCG2 expression presented as percentage of ABCG2-positive (ABCG2^+^) RBCs, means with SDs. (**B**) Representative flow cytometry histograms of PS exposure measured by annexin V in unstained MPN RBCs (black) and in MPN RBCs treated in vitro with DMSO (violet), ruxolitinib (blue), and KO143 + ruxolitinib (red). (**C**) Flow cytometry analysis of PS exposure measured using annexin V on MPN and *ABCG2_null_* RBCs treated with DMSO or 50 µM ruxolitinib for 72 h with or without 0.01 µM KO143 pre-treatment. Wilcoxon test, * *p* < 0.05, *** *p* < 0.001.

We next examined whether ruxolitinib, another important drug used in MPNs, is a substrate of ABCG2. It has been reported that ruxolitinib triggers phosphatidylserine (PS) exposure (an apoptotic marker) on RBCs from healthy individuals via p38 MAP kinase activation downstream of JAK2 [18]. Considering the clinical importance in PV, we addressed the ruxolitinib transport by ABCG2 in RBCs from 12 MPN patients with a range of ABCG2^+^ RBCs of 20–85% (Figure 3A) and from *ABCG2_null_* individuals as the control. MPN RBC samples treated ex vivo with ruxolitinib displayed high PS exposure levels measured by flow cytometry, with an average of 19% PS-positive RBCs (range: 10–30%) compared to DMSO-treated cells (15% PS-positive RBCs, range: 8–28%, *p* = 0.0005) (Figure 3B,C), suggesting the import of ruxolitinib into the RBCs. When MPN RBCs were pre-treated with the ABCG2 inhibitor KO143 prior to adding ruxolitinib, there was a significant decrease in PS-positive RBCs with an average of 16% (range: 6–25%, *p* = 0.021) (Figure 3B,C) showing that active ABCG2 potentiates ruxolitinib-induced PS exposure on MPN RBCs and suggesting that ruxolitinib influx is reduced when the ABCG2 transporter is blocked. Interestingly, *ABCG2_null_* RBCs treated with ruxolitinib displayed variable levels of PS-positive RBCs (Figure 3C), suggesting that another transporter may compensate for the absence of ABCG2 in these patients’ cells. As expected, there was no impact of KO143 on PS exposure in these cells (Figure 3C, *p* = 0.625), confirming the absence of ABCG2 and the specificity of this inhibitor.

### 2.4. ABCG2 Inhibition Reduces Ruxolitinib-Induced Apoptosis in K562 Cells

**Figure 4:**
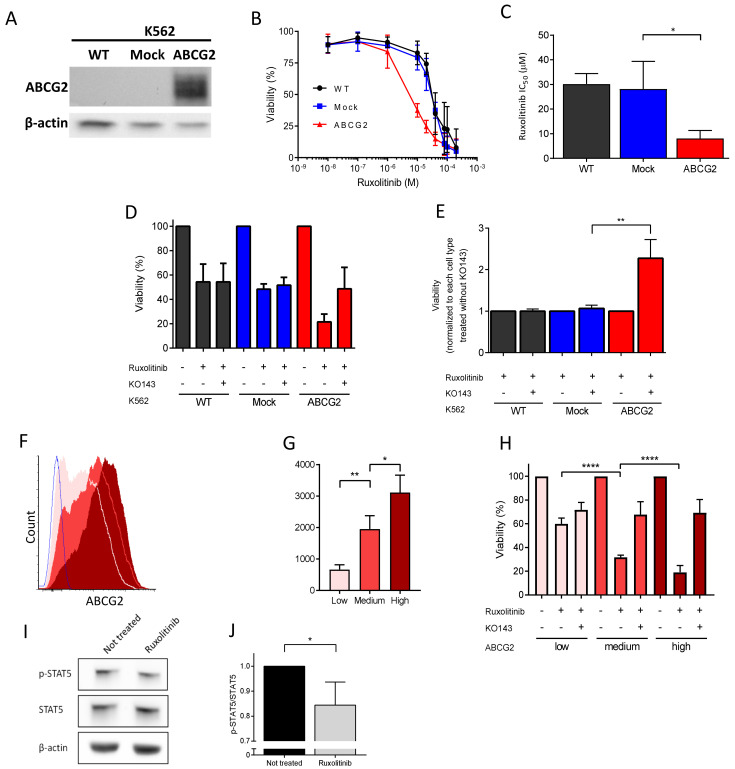
Ruxolitinib-induced apoptosis in K562 ABCG2 cells is dependent on the ABCG2 expression level and is reduced by ABCG2 inhibition. (**A**) SDS-PAGE immunoblots of ABCG2 expression on K562 WT, mock, and ABCG2-transfected cells. Beta-actin served as the loading control. (**B**) Ruxolitinib-induced cytotoxicity of K562 WT- (black), mock- (blue), and ABCG2-transfected cells (red). Cell viability was measured by CCK8 72 h after 0–200 μM ruxolitinib treatment. (**C**) Ruxolitinib IC_50_ values of K562 WT- (black), mock- (blue), and ABCG2-transfected cells (red). The IC_50_ values were calculated using a dose–response curve using Prism 7. Means with SDs, *N* = 4, Mann–Whitney test, * *p* < 0.05. (**D,E**) ABCG2 inhibitor KO143 rescues ruxolitinib-induced cytotoxicity of K562 ABCG2 cells. The viability of cells treated with 0 or 30 µM ruxolitinib (K562 WT IC_50_) for 72 h with or without 0.5 µM KO143 pre-treatment was measured by CCK8. The data is normalized to (**D**) not treated cells and (**E**) ruxolitinib-treated cells in the absence of KO143. Each condition was performed in triplicates. Means with SDs, *N* = 5, Mann–Whitney test, ** *p* < 0.01. (**F**) Representative flow cytometry histograms and (**G**) mean fluorescence intensity (MFI) of ABCG2 expression of sorted K562 ABCG2-transfected cells: low (pink), medium (red), high (maroon). ABCG2 mock-transfected cells (blue) served as the negative control. (**H**) Ruxolitinib-induced apoptosis in K562 ABCG2 cells is ABCG2 dose-dependent. Sorted K562 ABCG2 cells were treated with 30 µM ruxolitinib (K562 WT IC_50_) with or without 0.5 μM KO143 pre-treatment. The viability was measured by CCK8. The data are normalized to the viability of DMSO-treated cells as 100%. ABCG2, low (pink), medium (red) and high (maroon). Means with SDs, *N* = 4, Mann–Whitney test, * *p* < 0.05, ** *p* < 0.01, **** *p* < 0.0001. (**I**) SDS-PAGE immunoblots of phospho-STAT5 (*p*-STAT5) and STAT5 in K562 ABCG2 cells with and without ruxolitinib treatment. Beta-actin served as the loading control. (**J**) Quantification of the *p*-STAT5/STAT5 ratio from (**H**) in ruxolitinib-treated cells normalized to the *p*-STAT5/STAT5 ratio without treatment. Means with SDs, *N* = 4, Mann–Whitney test, * *p* < 0.05.

To further explore the ruxolitinib transport by ABCG2, we measured its impact on cell survival using a transfected K562 cell line expressing recombinant ABCG2 (Figure 4A). Ruxolitinib treatment blocks the JAK2/STAT5 signaling pathway leading to reduction of the cell count [19]. We treated K562 WT-, mock-, and ABCG2-transfected cells with 0–200 µM ruxolitinib over 72 h and measured their viability. The K562 ABCG2 cells displayed a significantly lower IC_50_ value of ruxolitinib compared to the mock-transfected cells (Figure 4B,C), indicating that increased ABCG2 levels enhance ruxolitinib-induced apoptosis. To further explore the hypothesis of ruxolitinib import by ABCG2, we treated the cells with ruxolitinib with or without pre-incubation with the ABCG2 inhibitor KO143. Pre-treatment with KO143 more than doubled K562 ABCG2 cell viability in the presence of ruxolitinib, while it had no effect on K562 WT- and mock-transfected cells (Figure 4D,E), suggesting that ABCG2 contributes to ruxolitinib import into the intracellular compartment. Furthermore, we sorted the K562 ABCG2 cells by flow cytometry gating on the 20% of the cell population expressing the lowest, highest, and intermediate mean fluorescence intensity of ABCG2 (Figure 4F,G). Incubating the three cell lines with 30 µM ruxolitinib showed an inverse relationship between ABCG2 expression levels and cell viability (Figure 4H), confirming the role of ABCG2 in enhancing ruxolitinib-induced apoptosis and further suggesting ruxolitinib import. Of note, pre-incubating the three cell lines with KO143 showed similar viability, suggesting that there are multiple ruxolitinib importers in these cell lines, which is in accordance with the results obtained with K562 WT- and mock-transfected cells. Finally, and as expected, ruxolitinib treatment led to a decrease in the phospho-STAT5 signal as determined by Western blotting using a specific anti-phospho-STAT5 antibody (Figure 4I,J), supporting ruxolitinib import within the intracellular compartment in these cells.

### 2.5. ABCG2 Inhibition Rescues Ruxolitinib-Induced Apoptosis during Human In Vitro Terminal Erythroid Differentiation

**Figure 5:**
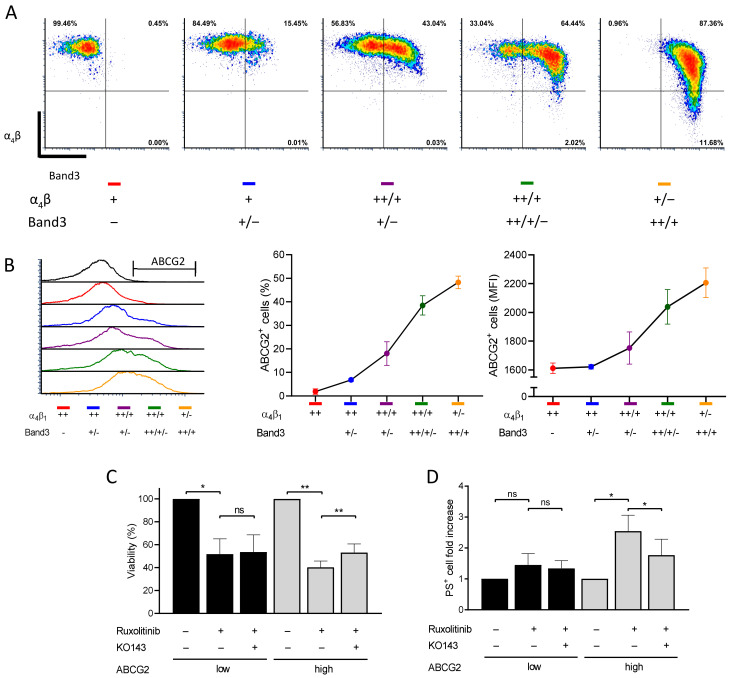
ABCG2 inhibition reduces ruxolitinib-induced apoptosis and PS exposure during human in vitro terminal erythroid differentiation. (**A**) Flow cytometry analysis of in vitro differentiated human erythroid cells. The in vitro cultured erythroblasts were labelled for GPA, α_4_ integrin, band 3, and 7AAD. Representative plots of α_4_ versus band 3 of all GPA-positive and 7AAD-negative cells are shown depending on the α_4_ and band 3 expression levels: ++ (high), + (low), − (negative). (**B**) Flow cytometry analysis of ABCG2 expression on the cell surface at different stages of in vitro human terminal erythroid differentiation. The ABCG2 levels are presented depending on the α_4_ and band 3 expression levels: ++ (high), + (low), − (negative). Left: ABCG2 histogram overlays; center: ABCG2-positive cells; right: mean fluorescence intensity (MFI) of ABCG2-positive RBCs. Means with SDs, *N* = 3. (**C**) Viability of in vitro differentiated human erythroid cells treated with 0.1 µM ruxolitinib with or without 1 μM KO143 pre-treatment. The viability was measured by CCK8. The data are presented at early (low ABCG2; black) and late (high ABCG2; grey) stages and are normalized with the viability of DMSO-treated cells as 100%. Means with SDs, *N* = 3, ratio paired *t*-test, ns – not significant, * *p* < 0.05, ** *p* < 0.01. (**D**) PS exposure at the surface of in vitro differentiated human erythroid cells treated with 0.1 µM ruxolitinib with or without 1 μM KO143. PS exposure was measured by flow cytometry using an annexin V kit. The data are presented at early (low ABCG2; black) and late (high ABCG2; grey) stages and are normalized with the PS exposure levels of DMSO-treated cells as 1. Means with SDs, *N* = 3, ratio paired *t*-test, ns – not significant, * *p* < 0.05.

*ABCG2* gene expression is induced during terminal erythroid differentiation as reflected by increasing ABCG2 mRNA levels [10]. However, ABCG2 protein levels during the late stages of erythropoiesis have not been studied. To address ABCG2 protein expression dynamics, we used a two-phase culture system to grow and differentiate human erythroid progenitors. Erythroid differentiation kinetics were assessed by flow cytometry following glycophorin A (GPA), integrin α4, and band 3 expression levels as previously described [20]. CD34^+^ cells isolated from three healthy donors were differentiated and displayed the expected kinetics of the three markers during the second culture phase (Figure 5A). ABCG2 was detected at the cell surface of differentiating erythroid cells in the α4^high^/band 3^neg^ population and increased continuously reaching a mean of 48.3% (SD = 2.7) of ABCG2-expressing cells in the α4^low^/band 3^high^ population (Figure 5A,B). The increase in ABCG2 expression was reflected by higher proportions of ABCG2-expressing cells and higher mean fluorescence intensity of ABCG2-positive cells. Because of the continuous ABCG2 upregulation, we speculated on increased ruxolitinib-induced cytotoxicity during terminal erythroid differentiation. Indeed, higher ruxolitinib-induced cytotoxicity was observed at late stages (high ABCG2 expression) compared with early stages (low ABCG2 expression) (Figure 5C). Moreover, pre-treatment with KO143 increased cell viability in ABCG2^high^ populations indicating that ruxolitinib-induced cytotoxicity was dependent on ABCG2 expression (Figure 5C). Finally, we assessed the effect of ruxolitinib in the presence or absence of KO143 on PS exposure at the cell surface as an apoptotic marker. Ruxolitinib-treated cells displayed an increased PS^+^ cell count during the late stage of terminal erythroid differentiation (Figure 5D). Furthermore, pre-treatment with KO143 partially inhibited ruxolitinib-induced PS exposure confirming that ABCG2 potentiates ruxolitinib-induced apoptosis during human in vitro terminal erythroid differentiation and further suggesting ruxolitinib import by ABCG2 in these cells (Figure 5D).

### 2.6. Analysis of Ruxolitinib Transport Ex Vivo and In Silico

**Figure 6:**
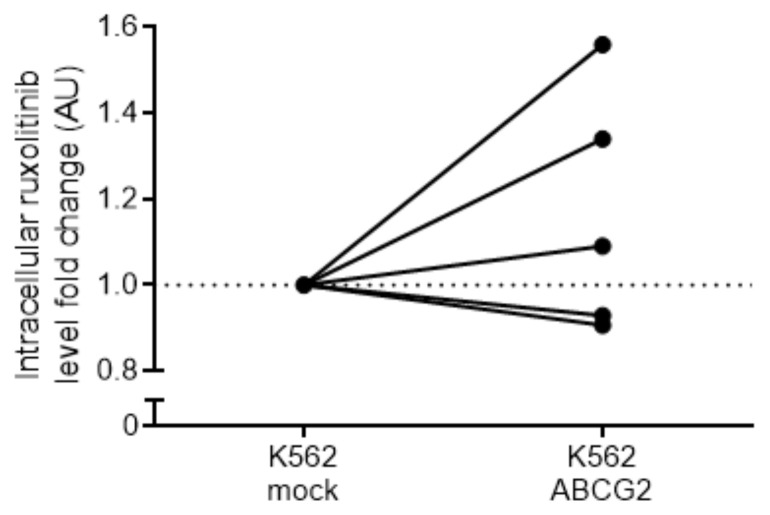
Analysis of intracellular ruxolitinib concentration in K562 ABCG2 cells. K562 mock- and ABCG2-transfected cells were treated with ruxolitinib and the intracellular ruxolitinib concertation was measured using LC–MS. Five independent experiments were performed in quadruplets for each cell type. Data are normalized by the average ruxolitinib signal of the mock-transfected cell line for each separate experiment. Untreated cells showed no detectable ruxolitinib signal.

We addressed direct ruxolitinib transport by ABCG2 using liquid chromatography–mass spectrometry (LC-MS) measuring intracellular ruxolitinib concertation in K562 mock and K562 ABCG2 cells. Three out of five repeats showed increased intracellular ruxolitinib concentration in K562 ABCG2 cells compared to K562 mock cells after 1 h of incubation with ruxolitinib (1.33-fold increase, SD = 0.24), suggesting direct ruxolitinib import by ABCG2 (Figure 6). However, two out of five repeats displayed a minor but opposite effect (1.08-fold decrease, SD = 0.02). Altogether, this method did not provide conclusive directionality of the ABCG2 transporter for ruxolitinib.

**Figure 7:**
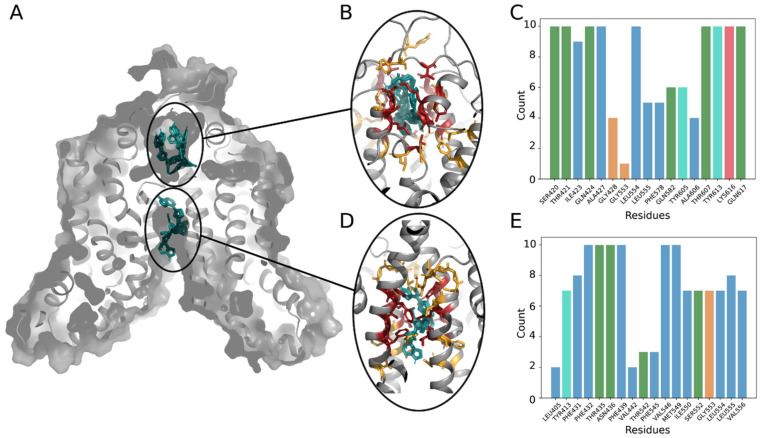
Ruxolitinib docking to the ABCG2 model. (**A**) General view of the ABCG2 dimers’ structure with two main binding sites (extracellular and intracellular cavities) and 10 best binding poses of a ligand (shown in cyan). (**B**,**D**) Close view at the main binding sites: residues contacting with a ligand in all 10 poses are colored in red, residues interacting with the ligand only in several positions are colored in yellow. (**C**,**E**) Number of interactions detected between ruxolitinib and residues in the 10 best positions. Bars are colored according to the standard Clustal X color scheme: blue for hydrophobic, green for polar, red for positively charged residues, orange for glycine, and cyan for tyrosine.

In order to provide atomistic details of the ruxolitinib transport by ABCG2, we developed a robust molecular model of the system. We performed long molecular dynamic simulations (for a total of 3 microseconds) on the different systems and defined possible ruxolitinib-binding site locations (see Materials and Methods for the details). We identified six representative conformations adopted by ABCG2 dimers during molecular dynamics simulations and used them together with the experimentally resolved structures for ruxolitinib docking. We detected two distinct ruxolitinib-binding sites (Figure 7A) both located in protein cavities buried in the transmembrane part of the protein and separated from each other by a hydrophobic barrier of leucine residues at positions 554 and 555. This dileucine motif was previously shown to act as a valve for drug translocation by ABCG2 [21] and thus must also play a crucial role in ruxolitinib transport by the protein. 

Hydrophobic residues are quite present in both binding pockets and are involved in ligand fixation. Indeed, at least one of the leucine residues is always in contact with the ruxolitinib molecule (Figure 7C,E). Moreover, in the intracellular cavity (Figure 7D), the majority of residues interacting with ruxolitinib are hydrophobic (Figure 7E). In the extracellular cavity, we also observed a number of polar residues surrounding the ligand in all the lowest energy binding poses (Figure 7B,C). The extracellular cavity also demonstrated lower values of binding energy estimated during docking (−8.68 ± 0.09 against −8.39 ± 0.07 kcal/mol), thus suggesting higher affinity of the ruxolitinib-binding sites as compared to the binding sites located in the larger intracellular cavity. Significantly buried ligand location, contact of the ruxolitinib molecule with the barrier-forming L554 and L555 residues, and similar orientation of the molecule in both binding sites (Figure 7B,D) suggest a possibility of ligand translocation between the sites (the minimal distance between ruxolitinib centers of mass between poses from different cavities in our model falls below 1.53 nm). Finally, the inhibitory effect of KO143 is in accordance with our previous observations and plugging of the intracellular cavity by KO143 is expected to block the ruxolitinib transport and lead to the experimentally observed effect in our ex vivo assays.

## 3. Discussion

Our results show increased ABCG2 levels on the PV RBC surface with further increase of these levels for patients undergoing HU treatment. ABCG2 increase on non-treated PV RBC membranes is not surprising as *ABCG2* is a *p*-STAT5 target gene [23,24] and as the STAT5 pathway is activated downstream of JAK2V617F in the erythroid lineage of PV patients. There was no correlation between ABCG2 levels on the RBC surface and the *JAK2V617F* allele burden, likely because allele burden measurements are performed with the DNA extracted from granulocytes, which does not reflect the allele burden of the erythroid lineage. Our stopped-flow experiments showed no difference in HU transport in RBCs despite high ABCG2 protein level differences between samples, indicating that HU is not a substrate of ABCG2 and suggesting that HU-driven ABCG2 increase is probably not related to drug resistance mechanisms. Of note, it is not surprising to detect HU-induced modulation of protein expression as, besides inhibiting DNA synthesis, HU has been shown to increase the expression of several membrane proteins on PV RBCs, including adhesion proteins Lu/BCAM and CD147 [12]. In the same study, we reported HU-mediated downregulation of numerous cell surface proteins, including KCNN4 (Gárdos), the Ca^2+^-activated potassium channel [12]. HU-induced protein level modulation is not restricted to MPNs. HU is known to induce fetal hemoglobin expression and is used for this primary purpose in sickle cell disease (SCD). Indeed, HU increases nitric oxide levels causing soluble guanylyl cyclase activation, subsequently increasing cGMP levels and leading to the activation of the gamma globin gene [25]. HU also increases the expression of the Lu/BCAM adhesion protein on the surface of RBCs in SCD [26,27] and modulates cell signaling pathways by regulating key effectors such as phosphodiesterase 9 in neutrophils [28] and phosphodiesterase 4A in endothelial cells [29]. Altogether, HU modulates gene expression and protein levels in the cytoplasm and on the cell membrane in multiple cell types and pathologies. Nonetheless, the molecular mechanisms altering protein levels, including ABCG2, remain to be investigated.

Ruxolitinib is a widely used drug in PV and myelofibrosis patients where it must be imported in the intracellular compartment in order to block JAK2 phosphorylation. Nevertheless, the transport of ruxolitinib is poorly studied. ABC proteins act as nutrient importers [30,31,32] but have been linked with resistance to numerous drugs including ABCG2-mediated export of tyrosine kinase inhibitors (TKIs), such as imatinib and nilotinib [7]. In the present study, we challenge the directionality of ABC proteins in TKI transport. Our study is the first showing potentiating effect of ABCG2 on ruxolitinib-induced apoptosis and consequently suggests ruxolitinib cellular import by ABCG2. Interestingly, elevated ABCB1 transporter levels have been linked with increased sensitivity to an iron-chelating drug [33]. Although it is interesting to speculate on a similar phenomenon for ruxolitinib, it is unlikely to take place as ruxolitinib binds with high affinity to the ATP-binding site of JAK2, and no other binding partners or iron-chelating abilities have been documented for this drug. Direct intracellular ruxolitinib measurements using LC–MS did not suggest conclusive ABCG2 transporter directionality for ruxolitinib, which can be explained by fast ruxolitinib transport kinetics and ruxolitinib metabolism within the cells. Importantly, our in silico analysis identified two distinct ruxolitinib-binding sites in ABCG2, further suggesting ruxolitinib transport by ABCG2.

Our study opens new avenues in the field of ruxolitinib efficacy research by defining specificity of targeted cell types depending on ABCG2 expression at the cell surface. Proerythroblasts from healthy individuals display low ABCG2 levels with continuous increase as the cells progress to a more mature state. This is in line with previously reported increasing ABCG2 mRNA levels during erythroid differentiation [10]. Increased ABCG2 levels in cultured erythroblasts are in accordance with elevated ruxolitinib induced cytotoxicity. Partial inhibition of this cytotoxicity by KO143 indicates that there are multiple ruxolitinib transporters. A recent clinical study revealed that the combination of HU and ruxolitinib results in high clinical response rates [34], probably because targeting multiple pathways has a better clinical effect than using these drugs separately. Nevertheless, a potential contributing factor to this better outcome would be HU treatment increasing the efficacy of ruxolitinib by improving its cellular import through increased levels of ABCG2 or similar transporters.

Although ruxolitinib reduces *JAK2V617F* allele burden, no complete molecular remission has been observed in patients treated with this inhibitor [35]. This can be partially explained by our study showing low ABCG2 expression level in common myeloid progenitors and the preferential targeting by ruxolitinib of the late stages of terminal erythroid differentiation where ABCG2 levels are higher. Therefore, it would be interesting to focus future studies on ruxolitinib efficacy in relation with ABCG2 expression in patient erythroid cells. In addition, a diversity of ABCG2 polymorphisms have been reported, some of which are associated with efficacy of statin drugs [36,37,38,39,40]. Consequently, ABCG2 expression levels along with a potential presence of ABCG2 polymorphism could serve as a marker for ruxolitinib efficacy in MPN patients.

## 4. Materials and Methods

### 4.1. Blood Samples

RBC samples from MPN patients and *ABCG2_null_* donors were cryopreserved at −196 °C at the National Reference Center for Blood Groups (CNRGS, biocollection #DC-2016-2872) as previously described [41]. Cryopreserved RBCs were thawed and shipped in acCell Stab (Bio-Rad, Hercules, CA, USA). The cells were washed three times in PBS (ThermoFisher Scientific, Waltham, MA, USA) before each experiment.

### 4.2. Cell Lines

K562 wild-type (WT) and K562 cells transfected with pCEP5 (mock) and pCEP5-ABCG2 (ABCG2) were cultured as previously described [42]. Caco2, HEK293, and HT29 cell lines were grown in DMEM + Glutamax + pyruvate supplemented with 1% MEM Non-Essential Amino Acids Solution (ThermoFisher Scientific, Waltham, MA, USA) and 10% FBS (Dominique DUTSCHER, Brumath, France).

### 4.3. In Vitro Erythroid Differentiation

CD34^+^ cells were purified from peripheral blood of healthy individuals using a magnetic beads system (Miltenyi Biotec, Bergisch Gladbach, Germany). The cells were cultured using the two-phase culture system [20]. The base medium was composed of the Iscove’s modified Dulbecco’s medium, 2 mM L-glutamine, 1x PS (ThermoFisher Scientific, Waltham, MA, USA), and 15% BIT (Stemcell Technologies, Vancouver, BC, Canada). In the first phase (day 0 to day 6), CD34^+^ cells were cultured in the base medium with addition of 100 ng/mL stem cell factor (SCF), 10 ng/mL IL-3, and 100 ng/mL IL-6 (Miltenyi Biotec, Bergisch Gladbach, Germany). In the second phase (day 7 to day 21), IL-3 was omitted from the culture and replaced by 2 UI/mL erythropoietin (Eprex (epoetin Alfa); Janssen, Beerse, Belgium). The cell concentration was adjusted to 0.7–1 × 10^6^ cells/mL. The cells were cultured at 37 °C in the presence of 5% CO_2_.

### 4.4. Western Blots

Erythrocyte membranes were prepared from MPN RBCs by hypotonic lysis performed for 5 min in an ice-cold 5P8 buffer (5 mM NaH2PO4; 0.35 mM EDTA, pH 8.0, containing 0.2 mM phenylmethylsulfonyl fluoride). Samples were centrifuged at 36,000× g at 4 °C for 30 min and washed several times with the 5P8 buffer until the supernatant was clear.

K562 ABCG2-transfected cells were treated with 0 or 30 µM ruxolitinib (Selleckchem, Houston, TX, USA) for 30 min. Samples were lysed for 45 min at 4 °C in a lysis buffer containing 20 mM Tris, 150 mM NaCl, 5 mM EDTA, 0.002% NaN3, 1% Triton X-100, 0.2% BSA, and phosphatase (Sigma-Aldrich, St. Louis, MO, USA) and protease inhibitor cocktails (Roche Diagnostics, Basel, Switzerland). Lysates containing the Laemmli buffer and 5% β-mercaptoethanol (Sigma-Aldrich, St. Louis, MO, USA) were size-separated in 8% SDS-PAGE and transferred on a nitrocellulose membrane (Whatman-Protan, Dassel, Germany). Anti-ABCG2 antibody (Santa Cruz Biotechnology, Dallas, TX, USA), anti-phospo-STAT5 and anti-STAT5 (Cell Signaling, Danvers, MA, USA), anti-mouse HRP-conjugated antibody (Abliance, Compiegne, France), and streptavidin-biotinylated HRP were used (GE Healthcare, Chicago, IL, USA). The signal was revealed by an ECL substrate with a ChemiDoc MP system and quantified using Image lab 6.0 (Biorad, Hercules, CA, USA).

### 4.5. Stopped-Flow Assays

The HU permeability of RBC variants was determined by using a stopped-flow spectrophotometer (SFM400, BioLogic, France) at 21 °C. Equal volumes of solutions A and B were used to mix RBCs (1% hematocrit in a PBS buffer) with a HU solution. For inhibition experiments, dimethylurea (DMU) (Sigma-Aldrich, St. Louis, MO, USA), a specific urea transporter B (UT-B) inhibitor [17], was added to solutions A and B at the same concentration. Time courses of the 90°-scattered light intensity (λ_exc_ of 530 nm) changes of RBCs were measured to follow the cell volume kinetics. Data from three time courses were averaged and fitted to single exponential functions by using the Simplex procedure of the BIOKINE software (BioLogic, Seyssinet-Pariset, France). The solute permeability coefficients (P_s_, cm/s) were calculated using the following equation: P_s_ = k_exp_ × V/S, where V/S is the volume-to-surface ratio and k_exp_ is the exponential time constant fitted to the erythrocytes swelling phase of light scattering which corresponds to solute entry.

### 4.6. Flow Cytometry

Erythroid cell differentiation was assessed using antibodies against glycophorin A (GPA), integrin α4 chain, band 3, and 7AAD with a BD FACSCanto™ II flow cytometry system (BD Biosciences, Franklin Lakes, NJ, USA) as described before [20]. Phosphatidylserine (PS) exposure was measured using an Annexin V-FITC kit following manufacturer’s recommendations (BD Biosciences, Franklin Lakes, NJ, USA). The anti-ABCG2 PE antibody was purchased from Biolegend (San Diego, CA, USA). K562 ABCG2 cells expressing low, medium, and high levels of ABCG2 were sorted by flow cytometry using an MA900 Multi-Application Cell Sorter (SONY, Tokyo, Japan).

### 4.7. Cytotoxicity and Apoptosis Assays

K562 WT-, mock-, and ABCG2-transfected cells were treated with 0–200 µM ruxolitinib for 72 h to calculate the IC_50_ of ruxolitinib. To treat the K562 WT-, mock-, and ABCG2-transfected cells, 30 µM ruxolitinib (IC_50_ K562-WT), a potent and selective ABCG2 inhibitor at the concentration used, was used with or without 0.5 µM KO143 pre-treatment for 1 h (Sigma-Aldrich, St. Louis, MO, USA) [43,44]. MPN and *ABCG2_null_* RBCs were treated with 50 µM ruxolitinib for 72 h with or without 0.01 µM KO143 pre-treatment for 1 h. Cultured erythroid cells were treated with 0.1 µM ruxolitinib for 48 h with or without 1 µM KO143 pre-treatment for 1 h. DMSO (Sigma-Aldrich, St. Louis, MO, USA) was used as the solvent control. Cell viability was measured by Cell Counting Kit-8 (CCK8) (Sigma-Aldrich, St. Louis, MO, USA) and PS exposure was measured using an annexin V kit.

### 4.8. Ruxolitinib Measurements by LC–MS

K562 mock- and ABCG2-transfected cells were treated with 30 µM ruxolitinib for 1 h at 37 °C. The cells were centrifuged for 5 min at 4 °C, and dry cell pellets were stored at −80 °C. Metabolites were extracted as previously described [45]. The volume of the extraction solution was adjusted to the number of cells (1 mL per 2 × 10^6^ cells). The extraction solution was composed of 50% methanol, 30% ACN, and 20% water. After addition of the extraction solution, the samples were vortexed for 5 min at 4 °C and then centrifuged at 16,000× g for 15 min at 4 °C. The supernatants were collected and stored at −80°C until analyses. LC–MS analyses were conducted on a QExactive Plus Orbitrap mass spectrometer equipped with an Ion Max source and a HESI II probe and coupled to a Dionex UltiMate 3000 UPLC system (ThermoFisher Scientific, Waltham, MA, USA). Each sample was injected in a HSS T3 column (100 mm × 2.1 mm i.d. 1.8 μm) (Waters Corporation, Milford, MA, USA) in the amount of 5 μL for liquid chromatography separation. Buffer A was 0.1% formic acid in water; buffer B, 0.1% formic acid in acetonitrile. The chromatographic gradient was run at a flow rate of 0.300 μL/min as follows: 0–5 min—0.1% B; 2–5 min linear gradient from 0.1% to 65% B; 5–25 min linear gradient to 99.9% B. The mass spectrometer was operated in the full-scan, positive polarity mode with the spray voltage set to 2.5 kV, the heated capillary held at 320 °C. The sheath gas flow was set to 20 units, the auxiliary gas flow was set to 5 units, and the sweep gas flow was set to 0 unit. The metabolites were detected across a mass range of 75–1000 m/z at a resolution of 35,000 (at 200 m/z) with the AGC target at 106 and the maximum injection time at 250 ms. Lock masses were used to ensure mass accuracy below 5 ppm. Data were acquired with the Thermo Xcalibur software (ThermoFisher Scientific, Waltham, MA, USA). The peak areas of ruxolitinib were determined using the Thermo TraceFinder software (ThermoFisher Scientific, Waltham, MA, USA), identified by the exact mass of a singly charged ion and by the known retention time on the HPLC column.

### 4.9. Molecular Dynamics of ABCG2 in Membrane Environment

We used the ABCG2 Cryo-EM structure (PDB ID: 6eti) as initial conformation for molecular dynamics (MD) simulations. The structure was resolved in the presence of the KO143 derivative (MZ29) acting as an inhibitor of ABCG2 efflux by plugging the lower cavity from the intracellular site [46]. We removed ligands and the nucleotide binding domain (NBD) from the initial structure and used the transmembrane domain of the protein (TMD) to build a membrane system. The TMD position in the lipid membrane was obtained using the OPM database. The protein was inserted into the POPC bilayer using the CHARMM-GUI utility [47] and solvated with addition of 0.15 M concentration of NaCl neutralizing system charge. We used the CHARMM36 force field [48] for model parametrization and performed MD simulations in an explicit solvent (type 3p [49]) using GROMACS 2016.4 [50]. Optimization of the model system geometry was performed using the steepest descent algorithm of energy minimization (10,000 steps) keeping positions of the heavy atoms of the protein fixed by harmonic potential (force constant of 1000 kJ/mol/nm). The resulting system was heated to the temperature of 310 K for 1 ns in the NVT ensemble using the Berendsen algorithm. Then, we performed a 50 ns equilibration simulation in the NPT ensemble restraining heavy atom positions for the protein molecule. The equilibrated system was used as the initial state for three replicates of production runs of 1 µs each (3 µs of simulation in total) to sample protein conformational transitions. During the simulations, we constrained covalent bond lengths and used an integration step of 2 fs. Equilibration and production runs were performed at normal pressure maintained by the semi-isotropic coupling using a Parrinello–Rahman barostat (coupling constant τ_P_ = 5.0 ps) and temperature controlled using the Nose–Hoover algorithm (coupling constant τ_T_ = 0.5 ps). All the simulations were performed at the temperature of 310 K with separate temperature coupling for the protein, lipids, and solvent molecules. We used periodic boundary conditions. The long-range electrostatic interactions were treated using the particle mesh Ewald (PME) method. The Lennard–Jones potential with a cut-off distance of 1.2 nm was used to describe non-bonded interactions.

### 4.10. Trajectory Analysis

**Figure 8:**
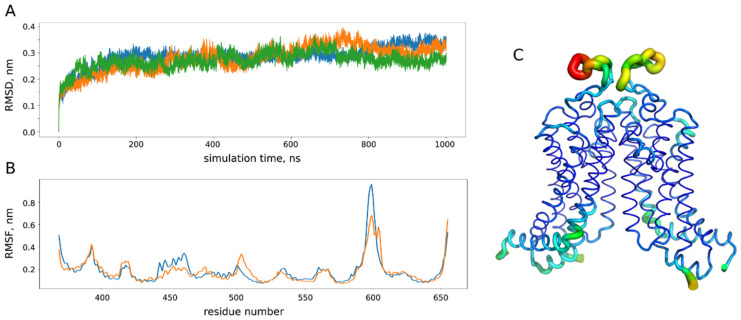
Analysis of molecular dynamics (MD) trajectories for the ABCG2 model. (**A**) Root mean square deviation (RMSD) of the protein backbone atom from the initial crystal structure (PDB ID: 6eti) in three replicates (blue, orange and green) of microsecond MD simulations. (**B**) Root mean square fluctuations (RMSF) of the protein backbone in three simulations (orange and blue correspond to different chains of a dimer). (**C**) RMSF projection on the protein structure: the most flexible parts are shown in yellow and red. During the simulations, we did not observe any pronounced conformational changes with the root mean square deviation from the initial experimental structure reaching approximately 3 nm after 100 ns of simulation, which is in accordance with 200 ns MD studies performed for ABCG2 homology models [22]. The most flexible part according to analysis of the protein backbone fluctuations corresponds to the extracellular loops (residues 580–610 in (**B**,**C**)), while the rest of the protein structure remains quite stable.

**Figure 9:**
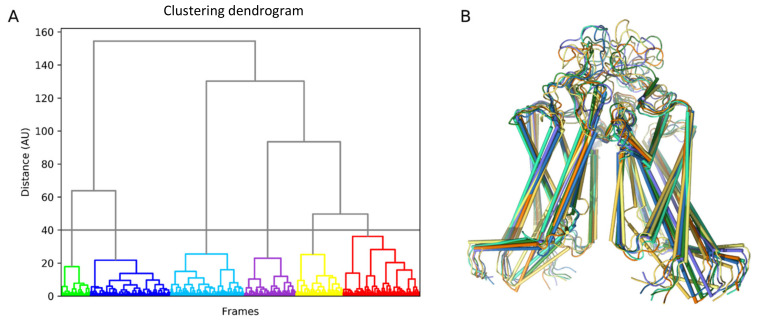
Clustering of ABCG2 conformations obtained in the molecular dynamics (MD) simulation. (**A**) Ttclust output for clustering results in the form of a dendrogram. (**B**) Corresponding cluster representative for the six conformers further used for ruxolitinib docking.

Trajectory analysis was performed using the standard GROMACS utilities and home-made Python scripts (Figure 8). In order to identify the representative conformations of the ABCG2 model, we clustered 3 µs of the MD trajectory using the ttclust software [51] with ward linkage (Figure 9).

### 4.11. Ruxolitinib Docking Procedure

The identified ABCG2 conformers together with crystal structures (PDB IDs: 6eti and 6hbu) were used to dock a ruxolitinib molecule. PDBQT files were generated using AutoDockTools v1.5.6 for further utilization in AutoDock VINA v1.1.2 [52]. The binding sites were identified using a docking box covering the whole transmembrane domain (with dimensions of 78 × 58 × 90 Å^3^) and increased exhaustiveness parameter (equal to 20) to compensate for the large search space volume. For each of the eight conformers, we found 20 poses and repeated docking procedure 20 times changing the random seed (3200 docking poses in total). Once two main binding sites were determined, ruxolitinib docking was performed to each binding pocket individually using a docking box of 20 × 20 × 30 Å^3^ in order to make our results more precise. For each pocket, ten best docking poses were chosen among all the conformers and all the docking runs. These poses were used for further analysis of residues involved in ligand binding.

### 4.12. Statistics

The data are presented as the mean values with SDs (Prism 7, GraphPad, San Diego, CA, USA); *p* < 0.05 was considered statistically significant.
